# Characterization of Cellobiose Dehydrogenase from a Biotechnologically Important *Cerrena unicolor* Strain

**DOI:** 10.1007/s12010-015-1667-2

**Published:** 2015-05-24

**Authors:** Justyna Sulej, Grzegorz Janusz, Monika Osińska-Jaroszuk, Patrycja Rachubik, Andrzej Mazur, Iwona Komaniecka, Adam Choma, Jerzy Rogalski

**Affiliations:** Department of Biochemistry, Maria Curie-Skłodowska University, Akademicka 19 St., 20-033 Lublin, Poland; Department of Genetics and Microbiology, M. Curie-Skłodowska University, Akademicka 19 St., 20-033 Lublin, Poland

**Keywords:** Cellobiose dehydrogenase, *Cerrena unicolor*, Purification, Gene, Fungi

## Abstract

Cellobiose dehydrogenase (CDH), a secreted flavocytochrome produced by a number of wood-degrading fungi, was detected in the culture supernatant of a biotechnologically important strain of *Cerrena unicolor* grown in a modified cellulose-based liquid medium. The enzyme was purified as two active fractions: *Cu*CDH-FAD (flavin domain) (1.51-fold) with recovery of 8.35 % and *Cu*CDH (flavo-heme enzyme) (21.21-fold) with recovery of 73.41 %. As CDH from other wood-rotting fungi, the intact form of cellobiose dehydrogenase of *C. unicolor* is a monomeric protein containing one flavin and one heme b with molecular mass 97 kDa and pI = 4.55. The enzyme is glycosylated (8.2 %) mainly with mannose and glucosamine residues. Moreover, the cellobiose dehydrogenase gene *cdh1* and its corresponding cDNA from the fungus *C. unicolor* were isolated, cloned, and characterized. The 2316-bp full-length cDNA of *cdh1* encoded a mature CDH protein containing 771 amino acids preceded by a signal peptide consisting of 18 amino acids. Moreover, both active fractions were characterized in terms of kinetics, temperature and pH optima, and antioxidant properties.

## Introduction

Fungi form an important group of microorganisms that have beneficial effects on the environment and human life. In forest ecosystems, they are mostly responsible for breakdown of abundant large biopolymers such as cellulose, hemicellulose, and lignin [1]. White-rot basidiomycetes are a group of fungi comprising from 1600 up to 1700 species characterized by the ability to depolymerize and mineralize lignin using a set of extracellular ligninolytic enzymes and low molecular compounds [2, 3]. At the same time, in modern biotechnology, filamentous fungi are major sources of bioactive metabolites, including proteins, peptides, glycoproteins, polysaccharides, lipopolysaccharides, phenolic compounds, triterpenoids, lectins, lipids, and their derivatives [4].

Among the many hitherto-characterized fungal species, *Cerrena unicolor* was described in literature as one of the best laccase producers [5]. Moreover, this species belonging to *Aphyllophorales* was proved to secrete extracellular manganese peroxidase, versatile peroxidases [6], and xylanase or cellulase when grown on cellulose [7]. This fungus commonly called “mossy maze polypore” may be found on dead northern hardwood tree species as maple, birch, or alder, where it causes white rot [8]. Besides extracellular enzymes, *C. unicolor* may be a source of polysaccharides [9] or low molecular fractions of secondary metabolites [10], which possess interesting biomedical and bioelectrochemical properties. However, up to date, cellobiose dehydrogenase, which was proven a crucial enzyme in decomposition of both cellulose and lignin, has not been described in cultures of the genus *Cerrena*.

Cellobiose dehydrogenase (CDH; EC 1.1.99.18; cellobiose [acceptor] 1-oxidoreductase) is a fungal extracellular hemoflavoprotein, which was discovered in 1974 by Westermark and Eriksson in white rot fungi *Trametes versicolor* [11] and *Phanerochaete chrysosporium* (*Sporotrichum pulverulentum*) [12]. CDHs are usually monomeric enzymes that belong to the glucose–methanol–choline (GMC) family together with other sugar oxidoreductases like the catalytically related enzymes glucose oxidase, pyranose dehydrogenase, and pyranose-2 oxidase [13]. It is composed of two prosthetic groups, a heme type b (ferriprotoporphyrin IX) and a flavin adenine dinucleotide (FAD) [14] connected through a flexible polypeptide linker region enriched in hydroxy amino acids [15]. This enzyme catalyzes the oxidation of the reducing end of cellobiose and higher cellodextrins in vivo, whereas in vitro lactose and other oligosaccharides with β-1,4-glycosidic linkages are acceptable substrates [16]. The catalytic cycle of CDH involves oxidation of sugar substrates to corresponding 1,5-lactones using various electron acceptors with concomitant reduction of flavin to FADH_2_ [17]. Lactones are finally converted to their carboxylic acids, and flavin is reoxidized by the heme group in two single-electron steps reactions [18]. Phylogenetic analysis of all known *cdh* genes showed division of the enzymes into three distinct classes: class I, representing only basidiomycetous CDHs; class II, exclusively comprising ascomycetous CDHs; and class III, containing so far uncharacterized or actively expressed CDHs [19]. Although the physiological function of this enzyme has not yet been revealed, our current knowledge points to its participation in the degradation and modification of lignocellulose by generating hydroxyl radicals via the Fenton reaction [20]. Recently, an interaction of CDH with copper-dependent polysaccharide monooxygenases (PMOs) involved in the degradation of cellulose has been proposed [21, 22]. This model for oxidative cellulose degradation may be widespread throughout the fungal kingdom in parallel with the better described hydrolytic cellulase enzyme system [23]. Recent papers have reported successful application of cellobiose dehydrogenase in a large variety of bioprocesses such as biocatalysis, bioremediation, or production of lactobionic acid [24]. The unique catalytic and bioelectrochemical properties of CDH have been used in biosensors for detection of cellodextrins [25], maltose [26], lactose [27, 28], diphenolic compounds [29], and catecholamines [30] in biofuel cells [15, 31] or in biomedical applications [32, 33].

Given the widespread biotechnological application of cellobiose dehydrogenase, new sources of this enzyme are being constantly searched. Recently, *C. unicolor* strain FCL139 has been found to be a producer of laccase, a unique enzyme in many biotechnological applications. Hereby, we successfully attempted to purify and characterize cellobiose dehydrogenase from this strain. Moreover, the corresponding *cdh* gene and cDNA were sequenced and analyzed.

## Materials and Methods

### Microorganism and Culture Conditions

The white rot fungus *C. unicolor* was obtained from the culture collection of the Regensburg University and deposited in the fungal collection at the Department of Biochemistry (Maria Curie-Sklodowska University, Poland) under the strain number 139. The fungus was maintained on 4 % (*w*/*v*) malt agar plate. To obtain the inocula, pieces of agar plates with the fungus were grown in the Lindenberg and Holm [34] medium in conical flasks for 10 days at 25 °C. Ten-day-old mycelia were homogenized in a disperser homogenizer T18 basic ULTRA-TURRAX (IKA, Staufen, Germany). The fragmented mycelial culture (10 % *v*/*v*) was used as a standard inoculum for further studies.

In order to obtain the high level of CDH, the strain of *C. unicolor* (FCL139) was grown in submerged culture for 10 days on a cellulose-based medium [35] with authors’ modifications. The medium had the following composition (1 l): 5 g Avicel, 10 g (NH_4_)_2_HPO_4_, 1 g KH_2_PO_4_, 0.3 g MgSO_4_ × 7H_2_O, 0.08 g CaCl_2_, 5 mg ZnSO_4_ × 7H_2_O, 1.5 mg MnSO_4_ × 4H_2_O, 1.5 mg CoCl_2_ × 6H_2_O, 5 mg FeSO_4_ × 7H_2_O, 100 mg yeast extract, and 0.1 mg thiamine. The pH was adjusted to 6.5 with 5 M HCl. After inoculation, the cultures were incubated at 28 °C in an incubator shaker Multitron (Infors, Bottmingen, Switzerland) at 120 rpm.

### Enzyme Purification Procedure

The culture supernatant (6 l) was collected on day 10 from the cellulose medium after centrifugation (12,000×*g* for 30 min) on a 6K15 (Sigma, Osterode am Harz, Germany). The clear supernatant was concentrated to 300 ml by the Prep/Scale TFF Cartridge PTGC 10 k polyethersulfone (Millipore, Bedford, MA) and used as a source of a crude enzyme. The proteins in the crude preparation were precipitated by the addition of solid ammonium sulfate in the range of 15–85 % saturation. The resulting suspension was collected by centrifugation, and the protein pellet was resolved in 100 ml deionized water and desalted using a preparative chromatography column (8 × 30 cm) filled with a Sephadex G-50 carrier. Fractions containing the protein were concentrated and applied to an anion-exchange DEAE-Sepharose (fast flow) column (2.5 × 15) connected to Econo System (Bio-Rad, Richmond, VA). The column was previously equilibrated with a 50-mM sodium acetate buffer (pH 5.0), and the proteins bound on the chromatography matrix were eluted using a linear gradient of 0 to 0.5 M NaCl in the same buffer at a flow rate of 1 ml/min. Fractions containing cellobiose dehydrogenase activity were pooled and concentrated in an Amicon-stirred cell using a polyethersulfone membrane (10 kDa cutoff). In the next step, affinity chromatography was performed. The concentrated protein was loaded onto a lactose-CPG column (1.5 × 8 cm) equilibrated with buffer A (50 mM sodium acetate buffer (pH 5.5)), washed with buffer B (50 mM sodium acetate buffer (pH 4.0)), eluted with buffer C (200 mM sodium acetate buffer (pH 4.0)), and applied 0.7 M ammonium sulfate. The eluent was collected in 0.5 ml portions. Fractions containing CDH activity (obtained from lactose-CPG) were collected, and a chromatofocusing analysis was performed on an Econo-chromatography column (Bio-Rad, Richmond, VA, USA; 130 cm, packed to a bed height of 20 cm) with a Polybuffer exchanger PBE 94 equilibrated with 250 ml of 0.025 M imidazole-HCl buffer (pH 7.4). Samples from lactose-CPG chromatography showing CDH activity (5 ml) were injected onto the column, and the enzyme was desorbed by elution with 200 ml Polybuffer 74-HCl (pH 3.0) at a flow rate of 0.5 ml/min. The active fractions were pooled out, and the purified enzyme solutions were used for kinetic experiments.

### Synthesis of Lactose-CPG

The controlled porous glass (CPG) (Cormay, Lublin, Poland) was prepared according to the method described previously [36]. The support was activated by γ-aminopropyltriethoxysilane (γ-APTES) according to a method that permits a high density of amino groups on the glass surface [37]. The activated support (APTES-CPG) was further used for affinity chromatography by binding the CDH substrate lactose to the activated support according to a method described in detail elsewhere [38]. The resulting sorbent lactose-CPG was used in the affinity chromatography.

### Enzyme Assays and Protein Determination

The activity of cellobiose dehydrogenase was assayed according to Baminger et al. [1] with slight modifications. CDH activity was specifically determined by monitoring the reduction of the electron acceptor 2,6-dichloroindophenol (DCIP) (Sigma Chemical Co., St. Louis, MO, USA) at 520 nm (ε_520_ = 6.8 mM^−1^ cm^−1^), pH 4.5, and 30 °C using a Shimadzu UV-160A (Shimadzu, Tokyo, Japan) spectrophotometer. The reaction mixture (1 ml) contained 50 μl of 3 mM DCIP (solution in water containing 10 % v/v ethanol), 100 μl lactose (300 mM in 100 mM sodium acetate buffer, pH 4.5), 50 μl NaF (80 mM NaF) in water, and an appropriate amount of the same buffer. After temperature adjustment, the reaction was started by the addition of an appropriately diluted CDH sample (100 μl) and the decrease in absorbance was monitored during the first 60 s. The final enzyme activity was expressed as nkat per liter.

Alternatively, CDH activity was selectively determined by following the reduction of 20 μM cytochrome c at λ = 550 nm and 30 °C (Sigma Chemical Co., St. Louis, MO, USA). The reaction was performed in 100 mM sodium acetate buffer, pH 4.5, containing 30 mM lactose and 4 mM NaF. The extinction coefficient (ε) was 19.6 mM^−1^ cm^−1^ [39]. This assay determined the activity of the intact protein containing both the flavin and the heme domains.

The protein concentration was determined using the Bradford method [40] with crystalline bovine serum albumin (BSA) as a standard or by monitoring the ultraviolet (UV) absorbance at 280 nm.

### Spectral Characterization

The spectrum of CDH purified to homogeneity was recorded from 250 to 650 nm in both the oxidized and the reduced states using a Shimadzu UV-160A spectrophotometer (Shimadzu, Tokyo, Japan). Purified CDH was diluted in 100 mM sodium acetate buffer, pH 4.5, to an absorbance of ∼2.5 at 280 nm, and the spectrum was recorded before and immediately after the addition of an approximately 1000-fold molar excess of lactose to the cuvette. The index of purity (RZ) of the oxidized CDH was calculated as the ratio of the absorbance at 420 nm to the absorbance at 280 nm [19].

### Effect of Temperature and pH on CDH Activity and Stability

DCIP and cytochrome c as electron acceptors and lactose as a substrate were used for assessing the effect of pH and temperature on the cellobiose dehydrogenase activity and stability. The effect of pH on enzyme activity was estimated in the range from 2.5 to 8.0 in 0.1 M McIlvaine’s buffer. Dependence of stability on pH was determined at 30 °C by incubation in variable pH ranges (pH 2.0–9.0 0.1 M Britton-Robinson buffer) for 12 h followed by measurement of the residual activities every 30 min.

The optimum temperature of the purified CDH was determined by performing enzymatic assays at different temperatures (4–80 °C). The thermal stability was investigated by incubating the enzyme solution in a 0.1 M sodium acetate buffer (pH 4.5) at various temperatures (30–90 °C); aliquots were drawn every 30 min for 12 h, and their residual enzyme activities were measured. Controls were carried out using the enzyme solutions without preheating, and its activity was taken as 100 %.

### Determination of Kinetic Constants

Kinetic constants were determined for various concentrations of CDH substrates (0.1 to 10 mM) and DCIP and cyt c as electron acceptors. All measurements were performed in triplicates. The K_m_ and V_max_ for the purified enzyme were calculated by nonlinear least-squares regression, fitting the observed data to the Michaelis–Menten equation. The OriginPro 8 software (OriginLab Corporation, Northhampton, MA, USA) was used for data analysis.

### Effect of Metal Ions and Potential Inhibitors

Effects of various metal ions and other reagents on CDH activity were investigated by adding inorganic salts, imidazole, and SDS (to the final concentration from 0.1 to 100 mM) to the samples of the enzyme dissolved in 100 mM sodium acetate buffer (pH 4.5) to the total volume of 1 ml. These assays were performed with lactose as a substrate and DCIP as an electron acceptor. Control tests were performed in parallel in the absence of metal ions and inhibitors.

### Electrophoresis and Peptide Sequencing by LC-MS/MS

Sodium dodecylsulfate-polyacrylamide gel electrophoresis (SDS-PAGE) (10 %) was performed as described by Laemmli [41]. Proteins were visualized by silver staining [42] and Coomassie Brilliant Blue G250 using PageRuler Prestained Protein Ladder (Fermentas, Glen Burnie, MA, USA). After the electrophoretic separation of the samples, equal pieces of 2 × 7 mm were cut out from the gel lanes. The spectrometric analysis of polypeptides was carried out in the Environmental Laboratory of Mass Spectrometry, Institute of Biochemistry and Biophysics of the Polish Academy of Sciences in Warsaw (Poland). The equipment used was sponsored in part by the Centre for Preclinical Research and Technology (CePT), a project co-sponsored by European Regional Development Fund and Innovative Economy, The National Cohesion Strategy of Poland. The samples were analyzed by HPLC coupled with tandem mass spectrometry (liquid chromatography/two stage mass spectrometry - LC-MS/MS) according to Kordan et al. [39]. The output list of precursor and product ions was compared with the protein database of the National Center for Biotechnology (NCBI, USA) using the MASCOT local server.

### Analysis of the CDH Carbohydrate Moiety

For sugar analysis, the CDH sample was hydrolyzed with 2 M trifluoroacetic acid (TFA) (100 °C, 4 h). The liberated monosaccharides were reduced with NaBD_4_ and converted into alditol acetates [43]. The components were identified on the basis of retention times and mass spectra of authentic standards using the gas chromatography-mass spectroscopy technique. GC-MS was carried out on an Agilent Technologies gas chromatograph (7890A) connected to a mass selective detector (inert XL EI\CI MSD 5975C). The chromatograph was equipped with a capillary column HP-5MS (30 m × 0.25 mm, film thickness 0.25 μm) (Agilent Technologies, Santa Clara, CA, USA). The carrier gas was helium with a flow rate of 0.7 ml min^−1^. The temperature program was as follows: 150 °C for 5 min, raised to 310 °C at 5 °C min^−1^, and kept for 10 min. Total carbohydrates were determined by phenol–sulfuric method (Dubois) [44]. A standard curve was prepared to quantify mannose.

### Antioxidant Activity Assays

The antioxidant properties of *C. unicolor* CDH was investigated in the presence of cellobiose and lactose as the substrates of enzyme as we described previously [45]. The standards (Trolox and vitamin C) well known for their strong antioxidant activity were used as a positive control. All measurements were performed in triplicate.

### DPPH Free Radical-Scavenging Test

The antioxidant activity of cellobiose dehydrogenase was determined using the DPPH equivalent, according to an adapted colorimetric procedure described by Paduch et al. [46] with slight modification. This method is based on the ability of 1,1-diphenyl-2-picrylhydrazyl (DPPH), a stable free radical, to decolorize in the presence of antioxidants. The tested compound (0.1 ml) at concentrations ranging from 6.25 to 800 μg/ml was added to 0.1 ml of DPPH^.^ solution (0.2 mg/ml in ethanol). The absorbance was measured spectrophotometrically at 515 nm using a Microplate Reader Elx800 (BioTek, Winooski, VT, USA) after 15 min (the time required to achieve the reaction plateau) of incubation at room temperature.

The capability of scavenging DPPH^.^ radicals was calculated by the following formula:

$$ {\mathrm{DPPH}}^{.}\mathrm{scavenging}\ \mathrm{effect}\ \left(\%\right) = \left[\left({\mathrm{A}}_0-{\mathrm{A}}_1\right)/{\mathrm{A}}_0\right]\times 100 $$

where A_0_ means the absorbance of the control sample and A_1_ means the absorbance of the standards or tested compounds.

### ABTS Free Radical-Scavenging Test

The ABTS (2,2′-azinobis (3-ethylbenzothiazoline-6-sulfonic acid) diammonium salt) radical-scavenging ability of cellobiose dehydrogenase was recorded according to the procedure of Re et al. [47] with some modification. For detection of the antioxidant capacity, 10 μL of the investigated compounds at concentrations ranging from 6.25 to 800 μg/mL was mixed with 990 μL of the ABTS radical solution. The percentage of ABTS oxidation was calculated by the presented formula:

$$ {\mathrm{A}\mathrm{BTS}}^{.+}\mathrm{scavenging}\ \mathrm{effect}\ \left(\%\right) = \left[\left({\mathrm{A}}_0-{\mathrm{A}}_1\right)/{\mathrm{A}}_0\right]\times 100 $$

where A_0_ means the absorbance of the control samples and A_1_ is the absorbance at 734 nm of the investigated compounds/standards.

The EC_50_ value, defined as the amount of the antioxidant necessary to decrease the initial DPPH and ABTS concentration by 50 %, was calculated from the results. The inhibition curves were prepared, and EC_50_ values were obtained as described previously [10].

### DNA Manipulation Techniques

Standard techniques for plasmid isolation, agarose gel electrophoresis, and DNA cloning were employed [48]. Automatic sequencing was performed using the BigDye^TM^ Terminator Cycle Sequencing Kit and an ABI PRISM 310 sequencer or ABI PRISM 3730 XL (Applied Biosystems, Carlsband, CA, USA).

### Preparation of Total mRNA, cDNA Synthesis, and Amplification

Total mRNA and cDNA synthesis and amplification were performed, as described previously [45]. To amplify the CDH 3′ cDNA fragment, degenerate primer GSP1 was designed using CDH gene sequences available in GenBank. To amplify complete CDH cDNA, gene-specific primers (GSP) were designed on the basis of an available sequenced CDH 3′ cDNA fragment (Table 1).

**Table 1:** Gene-specific primer sequences and annealing temperatures

Primer	Sequence 5′–3′	Tm [°C]
CerCDHGSP1	GCCCAGTTWTCWTANGCWTCGAT	53.5–55.3
CerCDHGSP2	CGAAGGGTTGTCCGACACAGCCTGCCC	67.3
CerCDHGSP3	TCGACCGACGGCCAGCGCTACCTCG	67.5
CerCDHGSP4	GGGAGGTTCGCCGCCGCGGTG	66.1
genDNA1F	GCCCTGTTTCAGCTCTCC	52.6
genDNA1R	ACCGAAAGCATGATCTTTGAAGTCCG	58
genDNA2F	GGTGGACCAAGTACGGCTGAAAAG	59,1
genDNA2R	ATTGTCGAGATAACATCCTTGAGTGC	56,4

### Genomic DNA Isolation, Amplification, and Cloning of the cdh1 Gene

DNA from *C. unicolor* was isolated according to Borges et al. [49], as described previously [45]. To amplify the cellobiose dehydrogenase gene, two pairs of primers genCerCDH (Table 1) were designed on the basis of already sequenced CDH cDNA. All PCR amplifications were carried out using Sigma RedTaq in a Tpersonal thermal cycler (Biometra, Goettingen, Germany). Specific PCR products were purified using the Cleanup kit (A&A Biotechnology, Gdynia, Poland) and inserted into the pTZ57R/T vector from the InsTAclone kit (Fermentas, Glen Burnie, MA, USA). Clones with target fragments were analyzed by sequencing.

### Nucleotide Sequence Accession Numbers

The following GenBank accession numbers were given to the CDH nucleotide sequences determined in this study: KC862284—*C. unicolor* strain FCL139 cellobiose dehydrogenase gene (*cdh*), complete cds; KC862282—*C. unicolor* strain FCL139 cellobiose dehydrogenase mRNA (*cdh*), complete cds.

### Bioinformatics Tools

Nucleic acid sequences were analyzed using Lasergene v.8.0 analysis software (DNASTAR, Inc, Madision, WI, USA). Database searches were performed with the BLAST and FASTA programs at the National Centre for Biotechnology Information (Bethesda, MD, USA) and European Bioinformatics Institute (Hinxton, UK), respectively. Multiple DNA and protein sequence alignments were performed with the Clustal-W algorithm [50]. Phylogenetic tree visualization was performed using the TreeView applet [51]. Glycosylation sites were detected with NetNGlyc v.1.0 (http://www.cbs.dtu.dk/services/NetNGlyc/) and NetOGlyc v.4.0 [52]. Conserved domains were analyzed by CDART [53].

### Statistical Analysis

All presented results are expressed as a mean ± SD from three independent experiments (*n* = 3). The mean values as well as standard deviation were calculated by the Excel program (Microsoft Office 2010 package), and only values of *p* ≤ 0.05 were considered as statistically significant.

## Results and Discussion

### Production and Purification of Cellobiose Dehydrogenase

**Fig. 1:**
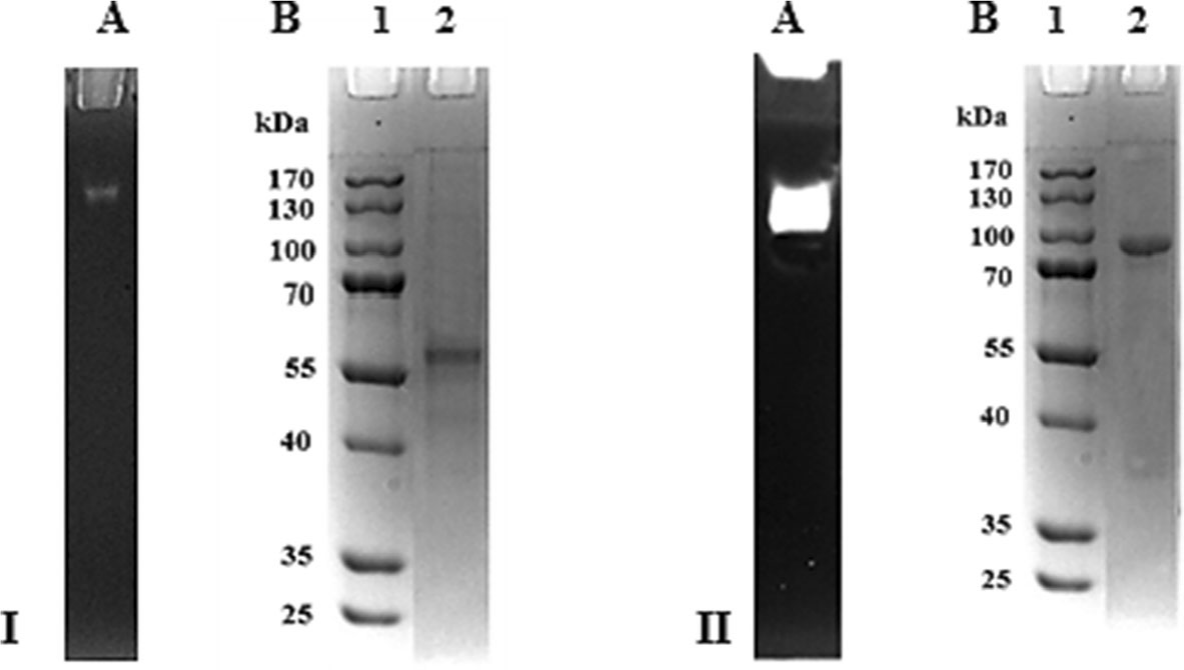
Activity staining (*A*) and SDS-PAGE (*B*) of the purified fractions cellobiose dehydrogenase from *Cerrena unicolor*: CuCDH-FAD (*I*) and CuCDH (*I*I). PageRulerPrestained Protein Ladder (Fermentas, Glen Burnie, MA, USA) (*lane 1*), purified CDH (*lane 2*)

Cellobiose dehydrogenase production by *C. unicolor* strain FLC139 was performed in shaking flasks on the cellulose-based medium. The mycelium was grown for 10 days, and then the culture liquid was collected, concentrated, and used as a source of crude enzyme for further purification steps and other studies. CDH was partially purified by ammonium sulfate precipitation in the range of 15 to 85 % saturation with a purification factor of 2.07-fold and a recovery of 84.93 % (Table 2). The resulting precipitate was dissolved in 100 ml distilled water and desalted on the Sephadex G-50 column. The protein fractions obtained from ammonium sulfate precipitation were applied to a DEAE-Sepharose chromatographic column. The elution profile from the ion-exchange chromatography on the DEAE-Sepharose column showed the CDH activity as a single peak, which was purified 28.97-fold with a yield of 70.34 %. The active fractions of cellobiose dehydrogenase were combined, concentrated on the stirred ultrafiltration cell equipped with a 10-kDa cutoff polyethersulfone membrane, and used in the subsequent step of chromatography on the lactose-CPG column. The affinity fractionation (lactose-CPG) gave only one cellobiose dehydrogenase activity peak purified approximately 53-fold in yields of 59 %. The active fractions of cellobiose dehydrogenase were pooled out and further fractionated by chromatofocusing on a Polybuffer exchanger PBE 94. A summary of the purification procedures of the cellobiose dehydrogenase is presented in Table 2. The last purification step (chromatofocusing) resulted in separation of two active fractions: *Cu*CDH-FAD (a flavin-only fragment of the enzyme), i.e., a very small fraction purified with recovery of 1.5 %, and *Cu*CDH (the intact enzyme) purified with a yield of 21.2 % (73.4-fold). Two CDH activities were also obtained from fungi *Irpex lacteus* [54], *T. versicolor* [55], and *Pycnoporus sanguineus* [45]; however, *Humicola insolens* contained three CDH fractions while *P. chrysosporium* contained only one [56]. In this study, the major fraction of CDH (*Cu*CDH) was used for further investigation as CDH from *C. unicolor*. The enzyme was purified from the culture supernatant to apparent homogeneity (Fig 1). The high spectral ratio A_420_/A_280_ (RZ value) is generally accepted as an indication of the absence of contaminating proteins [57]. The purified intact CDH from *C. unicolor* showed an A_420_/A_280_ ratio of 0.57, whereas the flavin fragment has a low RZ value (0.21). Besides the purity factor (RZ), the most important was the ratio of DCIP and cyt c activity (value around 1), which determines the rate of degradation of the intact enzyme on the flavin and heme domains [58]. The factor obtained in this study indicates that the intact CDH was rather stable in the culture conditions and during the purification procedure. The proteolytic cleavage to the DCIP active flavin fragment is negligible.

**Table 2:** Purification of CDH from *Cerrena unicolor* strain FCL139 culture filtrate

Purification step	Total protein (mg)	Total activity (nkat)	Specific activity (nkat/mg)	Yield (%)	Purification fold
Culture filtrate	1800.00	21180.00	11.77	100	1
Ultrafiltration (10 kDa)	1275.00	20377.50	15.98	96.21	1.36
Precipitation (NH_4_)_2_SO_4_	737.00	17988.00	24.41	84.93	2.07
DEAE-sepharose chromatography	43.70	14898.48	340.93	70.34	28.97
Lactose-CPG chromatography	20.30	12598.60	620.62	59.48	52.74
Chromatofocusing PBE-94 CuCDH-FAD	3.25	319.15	98.20	1.51	8.35
Chromatofocusing PBE-94 CuCDH	5.20	4491.50	863.75	21.21	73.41

### Enzyme Functional Parameters

**Fig. 2:**
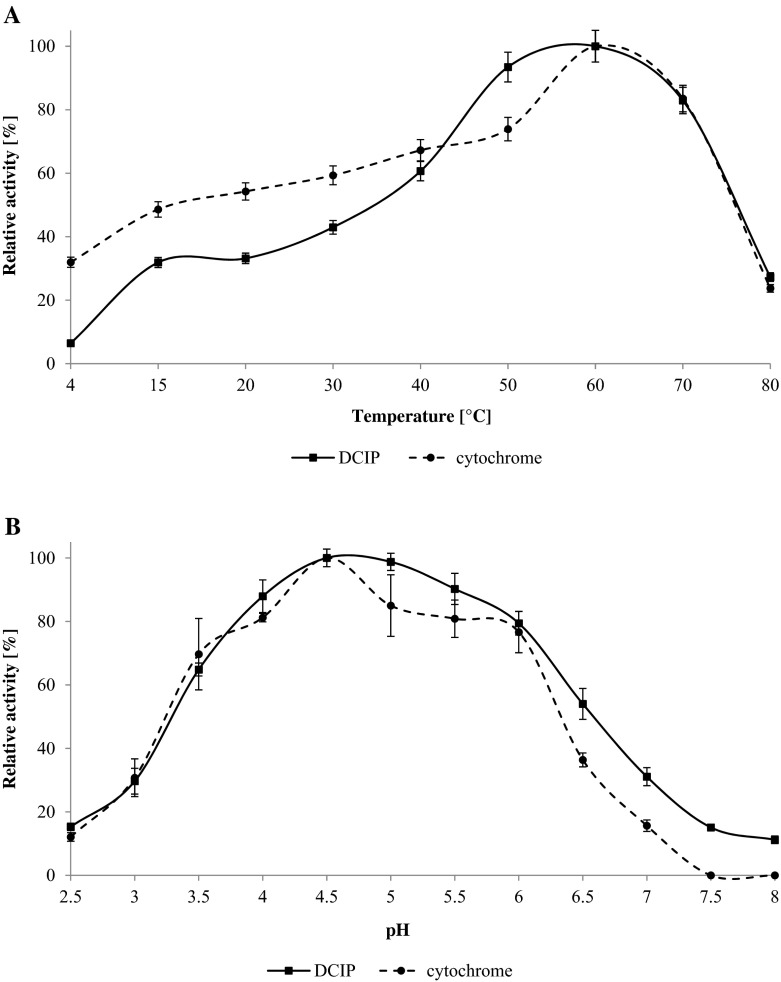
Effect of temperature (**a**) and pH (**b**) on activity of *Cerrena unicolor* CDH. Values represent the mean ± SD of triplicate samples

**Fig. 3:**
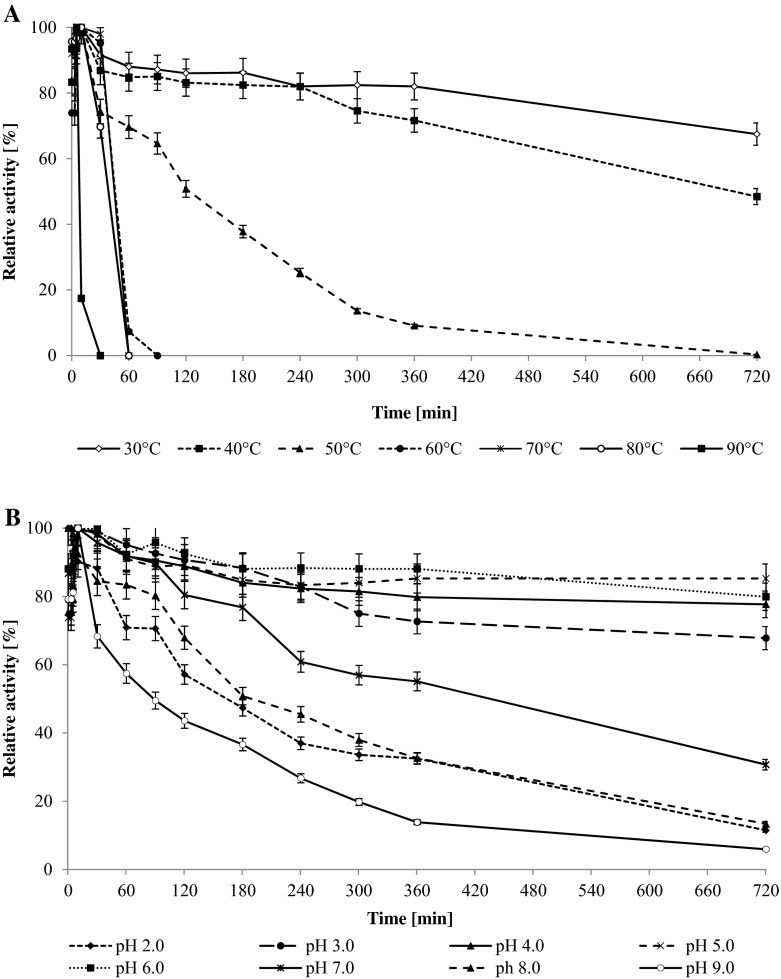
Thermostability (**a**) and pH stability (**b**) of *Cerrena unicolor* CDH. Activity was assayed with DCIP as the electron acceptor. Values represent the mean ± SD of triplicate samples

The optimum temperature of CDH was evaluated by measuring the activity of the purified enzyme at different temperatures. The enzyme activity was investigated with lactose as a substrate and two different electron acceptors (DCIP and cyt c). The maximum activity was recorded at 60 °C for both substances (Fig. 2A). Similar results were obtained for cellobiose dehydrogenase from *Ceriporiopsis subvermispora* [57]. Thermostability was examined by measurement of the activity over time (Fig. 3a). Complete loss of enzyme activity was recorded at 90 °C after heat exposure for 30 min and at 50 °C after 12 h. The enzyme seemed to be more stable than cellobiose dehydrogenase from *Pycnoporus cinnabarinus* [59].

The effect of pH changes on the activity of the purified enzyme from *C. unicolor* was also investigated using McIlvaine buffer with a pH range from 2.5 to 8.0. CDH exhibited maximum activity at pH 4.5 independently from the electron acceptor (Fig. 2B). Cellobiose dehydrogenases from most *Basidiomycetes* fungi are known to have pH optima in the acid range as opposed to *Ascomycetes* showing optimum activity in alkaline conditions [19]. CDHs from different sources are generally stable in a wide range of pH from 3 to 10 [15]. In this study, the enzyme was stable for 12 h at pH 3–6, losing 50 % of its initial activity within approximately 3 h at pH 2.0 and 8.0. CDH was especially sensitive to pH values above 9.0 (Fig. 3b).

The kinetic parameters of the purified cellobiose dehydrogenase were analyzed with cellobiose, lactose, and glucose as substrates using DCIP and cytochrome c as electron acceptors at 30 °C (Table 3). The results indicate that cellobiose was the best substrate of CDH with the catalytic efficiencies with a k_cat_/K_m_ value of 66 mM^−1^ s^−1^ when DCIP was an electron acceptor and 109 mM^−1^ s^−1^ when cytochrome c was used. Lactose was the least preferred substrate, with a k_cat_/K_m_ value of 4 and 11 mM^−1^ s^−1^ in the presence of DCIP and cytochrome c, respectively. The results obtained suggested that CDH from *C. unicolor* strain FCL139 was unable to oxidize glucose. Strong discrimination of glucose as a substrate is a characteristic for the Basidiomycete enzymes belonging to the class I CDHs [15]. Similar values of kinetic constants were reported for other fungal cellobiose dehydrogenases [60, 61].

**Table 3:** Kinetic constants of cellobiose dehydrogenase for carbohydrate substrates

Enzyme	Substrate	Electron acceptor	Km [mM]	Vmax [μM/min]	kcat [s^−1^]	kcat/Km [mM^−1^ s^−1^]
CuCDH-FAD	Cellobiose	DCIP	0.158	0.034	4.83	30.59
		Cyt c	–	–	–	–
	Lactose	DCIP	10.121	0.052	7.39	0.73
		Cyt c	–	–	–	–
CuCDH	Cellobiose	DCIP	0.285	0.189	18.86	66.18
		Cyt c	0.175	0.191	19.06	108.92
	Lactose	DCIP	5.241	0.209	20.86	3.98
		Cyt c	1.850	0.195	19.46	10.52

The influence of metal ions and substances that are potential inhibitors of different enzymes on the activity of cellobiose dehydrogenase was tested (Table 4). The activating/inhibiting effect of the analyzed substances on CDH was dependent on their concentration. The enzyme from *C. unicolor* is sensitive to higher concentrations of SDS and CuCl_2_, similarly to the protein from *P. sanguineus* [45]. Azide and cyanide have a slight inhibitory effect just like the CDH from *Schizophyllum commune* [61]. A similar activating effect in the case of divalent cations was observed by [40]. The other investigated reagents did not have any significant effect on the CDH activity.

### *C. unicolor* Cellobiose Dehydrogenase Structure

**Fig. 4:**
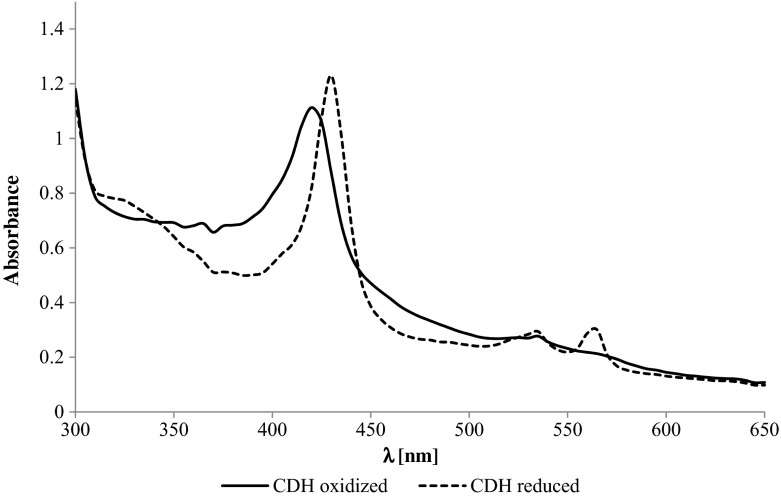
UV-visible spectrum of the oxidized (*line*) and reduced (*dashed line*) forms of CDH purified from *Cerrena unicolor* strain 139 in 100 mM sodium acetate buffer, pH 4.5. Reduction was performed with lactose. Spectra of oxidized (peak at 421 nm) and reduced (peaks at 429, 532, and 562 nm) CDH forms

The UV–vis spectra of the oxidized and reduced states of the purified CDH from *C. unicolor* indicated the presence of heme and flavin cofactors in the protein (Fig. 4). The main absorption peak of the oxidized enzyme appearing at 421 nm is typical for heme b, whereas the absorbance occurring in the region between 450 and 500 nm is mainly attributed to the FAD group [57, 62]. Reduction of the enzyme by addition of lactose resulted in appearance of peaks at 429, 532, and 562 nm and a decrease in absorbance at wavelengths between 450 and 500 nm, which probably represented the reduced form of FAD [62].

The molecular weight of both fragments (CuCDH-FAD and CuCDH) was predicted to be 58 and 97 kDa, respectively, as determined by SDS-PAGE analysis (Fig. 1). Most fungal CDHs are monomeric proteins with molecular masses between 80 and 115 kDa [15]. Native PAGE was also performed to identify the enzymatic activities of the proteins (Fig. 1).

Chromatofocusing was used to determine the isoelectric points for *Cu*CDH-FAD and *Cu*CDH, which were detected at pH 5.50 and 4.55, respectively. Many other intact CDHs from *Basidiomycetes*, such as *P. chrysosporium*, *I. lacteus*, *T. versicolor*, *C. subvermispora*, *Phlebia lindtneri*, and *P. sanguineus*, have acidic isoelectric points ranging from 3.0 to 5.1 [15, 45, 57, 63–65]. The protein containing only the FAD domain has a higher pI (5.5–6.7) [45, 63, 64].

Monosaccharide analysis of CDH from *C. unicolor* strain FCL139 showed that the sample contained mainly mannose (Man, 74.1 %). Small amounts of glucose (Glc, 6.2 %), galactose (Gal, 2.5 %), and glucosamine (GlcN, 17.2 %) were also present. Summarizing, the carbohydrate content of CDH was estimated at 8.2 % using the Dubois method with mannose as a standard. The up-to-date characterized fungal cellobiose dehydrogenases comprise from 8.9 up to 19 % of sugar moiety [66, 67].

**Fig. 5:**
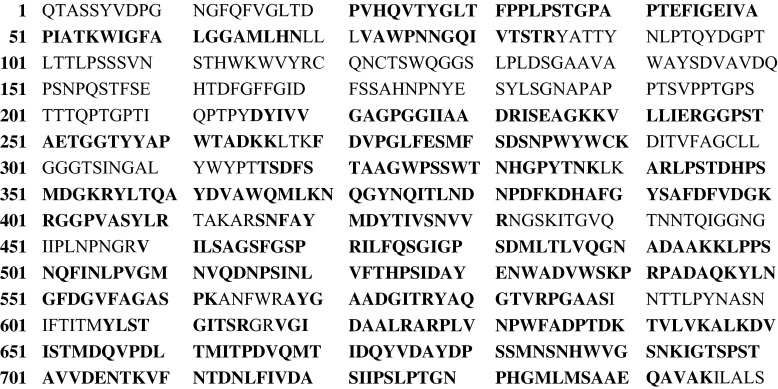
Complete sequence of *C. unicolor* CDH (AGS09133) with matched peptides in *bold* (sequence coverage: 67 %)

The identity of cellobiose dehydrogenase from *C. unicolor* was further proved by LC-MS/MS spectrometry analysis of the protein band observed in SDS-PAGE. The MS/MS raw data obtained were used to search against the NCBI protein database. The analyzed protein was identified when the MASCOT probability-based score (*p* < 0.05) was greater than 52. The protein from the gel slice was identified as CDH from *C. unicolor* with a MASCOT score of 55,248 and sequence coverage of 67 % (Fig. 5). The deduced molecular mass of *C. unicolor* CDH (80.9 kDa) was very similar to that determined by in silico analysis of the CDH amino acid sequence (82.6 kDa).

### Molecular Properties of *C. unicolor* CDH

**Fig. 6:**
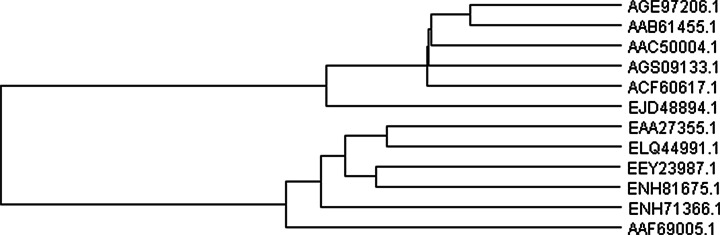
Unrooted UPGMA-based phylogenetic trees constructed with 12 protein sequences. The dendrogram of several cellobiose dehydrogenases from fungi. AGE97206- *Phlebia lindtneri*, AAB61455.1- *Phanerochaete chrysosporium*, AAC50004.1- *Trametes versicolor*, AGS09133- *Cerrena unicolor*, ACF60617.1-*Ceriporiopsis subvermispora*, EJD48894- *Auricularia delicata* TFB-10046 SS5, EAA27355.1- *Neurospora crassa* OR74A, ELQ44991.1- *Magnaporthe oryzae* Y34, EEY23987.1- *Verticillium alfalfae* VaMs.102, ENH81675.1- *Colletotrichum orbiculare* MAFF 240422, ENH71366.1- *Fusarium oxysporum f.* sp. *cubense* race 1, AAF69005.1- *Humicola insolens*

To our knowledge, this is the first in silico analysis of the *Cerrena* cellobiose dehydrogenase gene. Analysis of sequenced full-length cDNA of the *cdh1* gene from *C. unicolor* strain FCL139 revealed one open reading frame (ORF) of 2316 bp. The deduced protein sequence of *cdh1* shared similarity of 72 % with *P. lindtneri* cellobiose dehydrogenase (accession number AGE45679). The dendrogram obtained from the alignments of 12 cellobiose dehydrogenase amino acid sequences of *Basidiomycetes* and *Ascomycetes* showed that the putative CDH1 clustered together with a similar protein from *C. subvermispora* (Fig. 6). Moreover, both sequences belonged to the cluster including cellobiose dehydrogenases from *T. versicolor*, *P. lindtneri*, and *P. chrysosporium*. The analyzed protein sequence (771 aa) comprised a signal sequence within the first 18 amino acids (SignalP 0845). The results obtained place the length of the *C. unicolor* CDH signal peptide between *P. lindtneri* (17 aa) and *P. sanguineus* (19 aa) [45, 65]. A number of publications were produced indicating that proteolytic cleavage in the linker region resulted in the presence of the FAD domain in the culture medium [15, 68] even if some paper reported exceptions to this fact [57, 69]. However, our recent studies suggest that whether the linker region is digested by proteases may be a consequence of both its vulnerability and higher proteolytic activities in the cellulose-based medium. It is probable that among many proteases produced by white rot fungi only, one fraction is capable of cleaving cellobiose dehydrogenase. The problem should be addressed in detail in future studies comprising various techniques and fungal species.

Within the putative protein sequence of *C. unicolor* CDH, conserved domains typical for fungal cellobiose dehydrogenase were found at positions 23 to 189 aa (heme-binding cytochrome domain) from 206 to 231 aa (the linker region) and 233 to 769 aa (choline and flavoproteins domain). Moukha et al. [70] and Harreither et al. [19] proposed conserved residues constituting a putative cellulose binding module in basidiomycetous cellobiose dehydrogenases, which was also found in *P. sanguineus* CDH [45]. A similar module was detected in *C. unicolor* CDH in positions Tyr-275, Trp-279, Phe-288, Phe-294, Phe-298, Trp-304, Trp-306, and Phe-313. Analysis of *N*-glycosylation sites (Asn-X-Thr/Ser) showed glycosylation points at positions Asn-128, Asn-140, Asn-450, Asn-533, and Asn-615. In comparison with the *P. sanguineus* cellobiose dehydrogenase [45], only ten *O*-glycosylation sites were found within the linker region.

The complete *C. unicolor* CDH gene (3038 bp) was amplified by PCR using a genomic DNA as a template and primers designed on the basis of the nucleotide sequence of the *cdh1* cDNA, as described in the “Materials and Methods” section. The position of putative introns within the cellobiose dehydrogenase gene was determined by comparison of the genomic DNA and cDNA sequences. Eleven introns were found, ranging in size from 53 to 92 bp and all of them fell into the GT-AG rule [71]. Similarly to *P. sanguineus*, the cellobiose dehydrogenase gene contains fewer introns, likewise those of *P. cinnabarinus* [70] or *P. chrysosporium* [72]; however, the last intron is exceptionally long (92 bp).

### Antioxidant Activity Assays

**Fig. 7:**
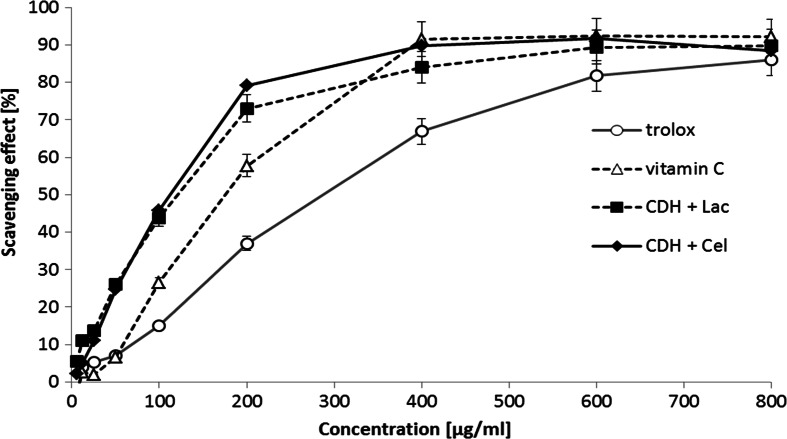
Comparison of the antioxidant properties of cellobiose dehydrogenase with and without substrates (cellobiose and lactose) from *Cerrena unicolor* determined by the ABTS method. Values represent the mean ± SD of triplicate samples

**Fig. 8:**
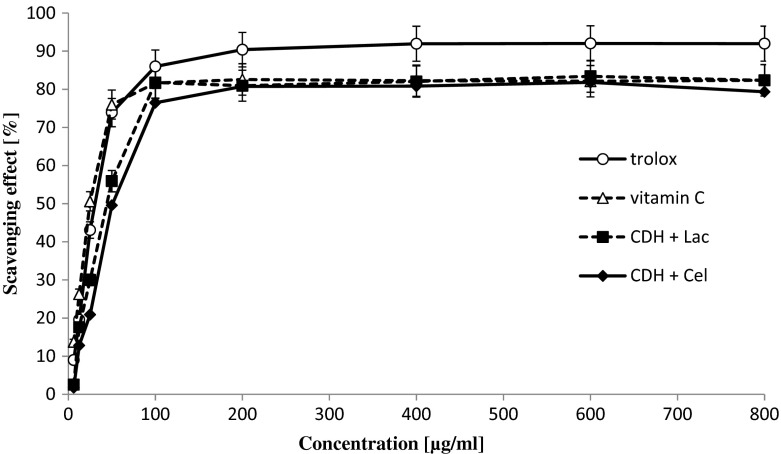
Comparison of the antioxidant properties of cellobiose dehydrogenase with and without substrates (cellobiose and lactose) from *Cerrena unicolor* determined by the DPPH method. Values represent the mean ± SD of triplicate samples

Fungi are producers of a large number of bioactive compounds with antioxidant properties. In the earlier studies, we have shown strong antioxidant capability of the fungal CDH from *P. sanguineus* [45]. Determination of the antioxidant activity of the newly isolated cellobiose dehydrogenase from *C. unicolor* is important for future research on its biotechnological potential. We applied two different methods commonly used to estimate the antioxidant potential: the DPPH and ABTS method. The obtained results showed that intact CDH without substrates have no antioxidant activity. Evaluation of antioxidant activity of the intact CDH with substrates (cellobiose and lactose) is shown in Fig. 7 (ABTS method) and Fig. 8 (DPPH method). Antioxidant activity measured by DPPH showed the similar effects as the ABTS method if the substrate was the electron donor. The scavenging abilities of CDH with lactose and cellobiose at the concentration range of 6.25–800 μg/ml were estimated at 89.3–91.6 % for ABTS and 81.8–82.1 % for the DPPH method, respectively. The EC_50_ values of CDH, i.e., the concentration of the enzyme necessary to decrease the initial concentration of DPPH and ABTS by 50 %, was calculated and expressed in Table 5. The lowest values of EC_50_ were observed for the DPPH radical scavenging method, i.e., 39.8 μg/ml for CDH with lactose and 48.5 μg/ml for CDH with cellobiose. The EC_50_ values for the ABTS method used for testing the CDH antioxidative properties were 103.7 and 93 μg/ml, respectively. All the results obtained indicate strong redox potential of the CDH enzyme only in the presence of lactose and cellobiose substrates. Therefore, it would be interesting to investigate whether *C. unicolor* CDH may be applied as an antimicrobial agent, as recently described for *Myriococcum thermophilum* cellobiose dehydrogenase [73].

**Table 4:** Effect of metal ions and some reagents on the cellobiose dehydrogenase activity

	Relative activity (%)					
	0.1 mM	1 mM	5 mM	10 mM	50 mM	100 mM
None	100 ± 0.00	100 ± 0.00	100 ± 0.00	100 ± 0.00	100 ± 0.00	100 ± 0.00
Imidazol	110 ± 0.73	109 ± 0.35	107 ± 5.31	102 ± 3.80	97 ± 0.01	67 ± 0.89
EDTA	100 ± 3.58	100 ± 2.11	98 ± 0.25	97 ± 2.48	91 ± 7.44	70 ± 8.57
NaF	100 ± 0.43	96 ± 3.73	93 ± 3.59	93 ± 1.29	58 ± 1.86	46 ± 4.88
KF	100 ± 4.41	99 ± 1.88	95 ± 1.81	89 ± 2.35	58 ± 0.29	49 ± 0.45
KCN	98 ± 1.74	98 ± 2.71	98 ± 0.77	97 ± 0.39	91 ± 0.58	33 ± 1.94
SDS	95 ± 2.41	71 ± 1.30	5 ± 0.01	3 ± 0.01	3 ± 0.56	0 ± 0.00
NH_4_Cl	102 ± 3.62	102 ± 1.50	102 ± 1.09	99 ± 1.67	97 ± 0.26	97 ± 1.42
Na_2_SO_4_	106 ± 5.57	109 ± 1.54	108 ± 8.42	101 ± 0.37	104 ± 0.77	105 ± 2.25
NaCl	94 ± 0.25	92 ± 0.53	94 ± 2.33	94 ± 2.26	97 ± 0.02	97 ± 1.56
KCl	102 ± 0.02	101 ± 0.32	100 ± 0.65	100 ± 3.30	103 ± 1.01	105 ± 2.02
MgCl_2_	99 ± 0.01	98 ± 1.40	98 ± 1.37	96 ± 1.15	87 ± 0.29	85 ± 1.46
CaCl_2_	99 ± 1.21	99 ± 0.61	98 ± 3.02	96 ± 2.24	94 ± 1.04	93 ± 0.58
CoCl_2_	105 ± 0.02	104 ± 0.84	102 ± 0.81	101 ± 0.81	102 ± 2.07	97 ± 0.22
MnCl_2_	100 ± 1.65	100 ± 1.43	100 ± 2.65	100 ± 2.36	99 ± 3.30	97 ± 3.32
CuCl_2_	98 ± 0.67	98 ± 1.54	93 ± 0.59	84 ± 2.20	7 ± 0.18	1 ± 1.15
ZnCl_2_	99 ± 5.11	99 ± 1.48.	99 ± 1.49	97 ± 2.10	95 ± 1.01	95 ± 1.65
MgSO_4_	101 ± 3.83	101 ± 0.89	101 ± 1.59	101 ± 0.01	100 ± 0.22	99 ± 1.79
MnSO_4_	102 ± 1.36	101 ± 3.19	103 ± 0.69	100 ± 0.45	100 ± 2.96	100 ± 0.91
CuSO_4_	101 ± 1.33	96 ± 2.41	96 ± 0.02	91 ± 2.80	86 ± 1.04	86 ± 0.60
ZnSO_4_	104 ± 1.12	103 ± 2.88	104 ± 3.11	100 ± 0.66	101 ± 0.22	102 ± 3.99

**Table 5:** EC_50_ values (effective concentration at which the radicals present in the investigated samples were scavenged by 50 %; the antioxidant activity was 50 %) of CDH isolated from *C. unicolor* submerged cultures in comparison to Trolox and vitamin C

	EC50 ($\mu$g/mL)	
	ABTS radical scavenging	DPPH radical scavenging
Trolox	251.6 ± 7.86	28.4 ± 0.72
Vitamin C	151.3 ± 5.14	25.1 ± 0.61
CDH	–	–
CDH + lactose	103.7 ± 3.76	39.8 ± 1.42
CDH + cellobiose	93.0 ± 2.53	48.5 ± 1.56

All results are expressed as mean ± SD from three experiments ($n$ = 3). Values within the column and the row for investigated samples are significantly different (*P* ≤ 0.05)

*C. unicolor* strain FCL139 was proven to produce not only biotechnologically important laccase but also cellobiose dehydrogenase with interesting features as well. Given the size and gene content of the available *C. unicolor* genome, its CDH is only part of the wood degrading machinery and it would be interesting to characterize in detail the abilities of this white rot fungus of decompose lignocellulose. The recent explosion of interest in cellobiose dehydrogenase, which was proven to act in concert with LPMO (lytic polysaccharide monooxygenase) in cellulose breakdown and was successfully applied in different biotechnological areas, encourages scientists all over the world to search for new CDH with exceptional features. By applying high throughput techniques, new insight into the wood decomposition is possible. In consequence, the acquired knowledge will contribute to accelerating the application of the discovered and characterized enzymes in new biotechnological areas.
